# Retrospective review of Google Trends to gauge the popularity of global surgery worldwide: A cross-sectional study

**DOI:** 10.1016/j.amsu.2021.102950

**Published:** 2021-10-16

**Authors:** Lorraine Arabang Sebopelo, Alexandre Jose Bourcier, Olaoluwa Ezekiel Dada, Gideon Adegboyega, Daniel Safari Nteranya, Ulrick Sidney Kanmounye

**Affiliations:** Research Department, Association of Future African Neurosurgeons, Yaounde, Cameroon

**Keywords:** Global health, Global surgery

## Abstract

**Introduction:**

Global surgery is a growing movement worldwide, but its expansion has not been quantified. Google Search is the most popular search engine worldwide, and Google Trends analyzes its queries to determine popularity trends. We used Google Trends to analyze the regional and temporal popularity of global surgery (GS). Furthermore, we compared GS with global health (GH) to understand if the two were correlated.

**Methods:**

This is a retrospective cross-sectional study examining Google Trends of GS and GH. We searched the terms “global surgery” and “global health” on Google Trends (Google Inc., CA, USA) from January 2004 to May 2021. We identified time trends and compared the two search terms using SPSS v26 (IBM, WA, USA) to run summary descriptive analyses and Wilcoxon rank-sum tests.

**Results:**

The ten countries most interested in GS were India (5.0%), the United Kingdom (5.0%), Ireland (4.0%), the United States (4.0%), Australia (3.0%), Canada (3.0%), New Zealand (3.0%), Germany (2.0%), South Africa (2.0%), and Nigeria (1.0%). GS became more popular after 2015 (2.3% vs. 1.3%, P < 0.001) and was consistently less popular than GH (1.6% vs. 45.3%, P = 0.04). The difference between GS and GH interest levels increased after 2015 (45.4% vs. 42.9%, P = 0.04).

**Conclusion:**

GS is less popular than GH, more popular in high-income countries, and has become more popular after 2015 when the Lancet Commission on Global Surgery published its seminal report. The World Health Organization passed resolution WHA 68.15. Future advocacy efforts should target low- and middle-income countries primarily.

## Highlights

• Global surgery is a growing movement worldwide, but its expansion and popularity has not been quantified yet.
• Google Search is the most popular search engine worldwide, and Google Trends analyses its queries to determine popularity trends.
• It is shown that the ten countries most interested in Global Surgery based on Google Trends are high income countries and this interest has grown since the publication of the Lancet Commission on Global Surgery report in 2015.
• The Global Surgery interest levels in low- and middle-income countries are still not proportional to the unmet surgical burden those countries are currently facing, so subsequent advocacy efforts should focus on equitable expansion of Global Surgery.

## Abbreviations

InciSioN: International Student Surgical Network
G4 Alliance: the Global Alliance for Surgical, Obstetric, Trauma, and Anesthesia Care
GH: Global health
GS: Global surgery
GSSA: Global Surgery Student Alliance
HICs: High-income countries
LCOGS: Lancet Commission on Global Surgery
LMICs: Low- and middle-income countries
SOTA: Surgical, obstetric, trauma, and anesthesia
WHO: World Health Organization

## 1 Introduction

Five billion people lack access to safe, timely, and affordable surgical, obstetric, trauma, and anesthetic (SOTA) care [1]. The unmet need for SOTA services is more pronounced in low- and middle-income countries (LMICs), where it increases morbidity and mortality from SOTA conditions. Global Surgery (GS) is an integrated approach to quality surgical care delivery, including anesthesia and public health worldwide, effectively and fairly [2]. In 2015, the Lancet Commission on Global Surgery (LCOGS) quantified the global burden of unmet SOTA diseases and proposed actions to increase access to SOTA care in LMICs [1]. One of the LCOGS’ key messages was that 33 million people were at risk of catastrophic health expenditures related to SOTA care [1]. The LCOGS proposed holistic interventions to strengthen health systems in under-resourced communities and developed targets for monitoring and evaluating these interventions [1]. In effect, the LCOGS provided a roadmap for the field of GS.

During the same year, the World Health Organization (WHO) passed Resolution WHA68.15 [3]. The resolution complemented the LCOGS report by promoting the integration of SOTA care into the emergency health care package [3]. Like the LCOGS report, the resolution highlighted the need to reinforce surgical systems in low-resource settings. It advocated for closing existing gaps between LMICs and high-income countries (HICs) [4]. Since its ratification, the resolution has successfully improved service delivery in under-resourced communities [3].

Multifaceted advocacy efforts have helped expand GS beyond the LCOGS and WHO. Multiple organizations have driven these efforts, including the Global Alliance for Surgical, Obstetric, Trauma, and Anesthesia Care (G4 Alliance). The G4 Alliance is composed of GS stakeholders from all over the world and different specialties working together to increase awareness, identify gaps, and propose solutions to GS problems [5]. Together, the members of the G4 Alliance push the GS at high-level meetings and lead grassroots efforts in low-resource settings.

The field of GS has grown rapidly through the development of subspecialties (ex: global anesthesia, global cardiac surgery, global neurosurgery, global obstetrics, global pediatric surgery, global trauma, and global urology) and research activities (i.e., academic GS) [6,7]. Indeed, medical students and trainees interested in surgical specialties and public health have found a home in GS. One organization contributing to the expansion of GS is the International Student Surgical Network (InciSioN) [4]. InciSioN, an active member of the G4 Alliance, is a medical student and trainee interest group with more than 5000 members worldwide [8].

Given the rise of initiatives focusing on GS and the lack of studies quantifying the expansion of this field, we wished to analyze the geographical and temporal expansion of GS worldwide by using Google Trends metadata. Google Trends is a tool that uses search information from Google Search to extract patterns and behaviors concerning queries [9]. Google Trends has previously been used to evaluate interest levels in epidemiology and clinical medicine [[9], [10], [11], [12], [13]]. Google Trends is also used to identify changes or updates on a topic of interest. It surveys networked patterns to highlight the temporal and regional popularity of a topic [13]. Therefore, the present study aimed to analyze the geographical and temporal expansion of GS worldwide by using Google Trends metadata to quantify interest levels. The study results should help identify target audiences for future GS advocacy and education initiatives.

## 2 Methods

We conducted a retrospective cross-sectional study of Google Trends for GS and GH. We searched the terms “global surgery” and “global health” in English, French, and Spanish from the inception of Google Trends (January 2004) to May 2021.

All procedures conformed to the guidelines of the Declaration of Helsinki and our study is registered in Clinical Trials Protocol Registration and Results System under the registration ID NCT05012085. https://clinicaltrials.gov/ct2/show/NCT05012085.

The work has been reported in line with the STROCSS criteria [14].

Ethical approval was not necessary for this study as it did not involve human or animal subjects, and the Google Trends database is open access.

We aggregated monthly interest level data by year and visualized the trends using bar charts. In addition, we aggregated GS monthly interest data temporally into two groups; a split decided to reflect the period before and after the LCOGS published its seminal report in 2015 [1].

We used SPSS v26 (IBM, WA, USA) to calculate the mean and 95% CI for the GS and GH interest levels. We equally calculated the difference in interest levels between GS and GH, and we evaluated the association between these variables using Spearman's correlation. Moreover, we used the Wilcoxon rank-sum test to evaluate the association between GS interest levels before and after 2015 and the difference in GS and GH interest levels before and after 2015.

## 3 Results

**Fig. 1:**
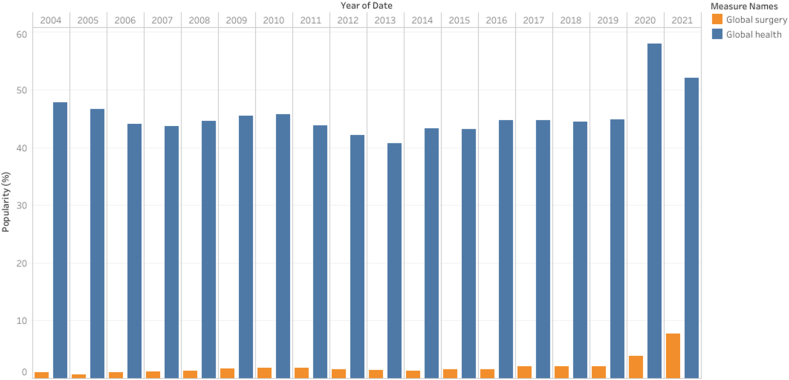
Popularity trends of global health and global surgery since 2004.

**Fig. 2:**
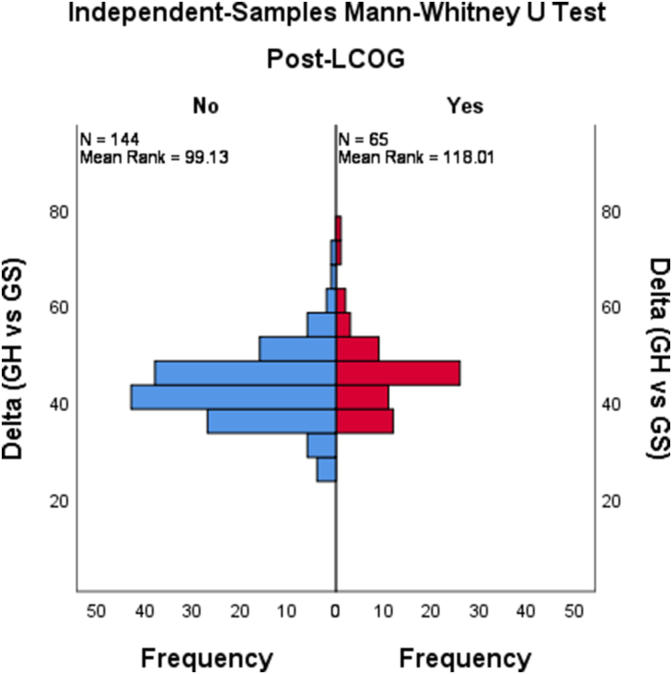
Difference in the interest levels of global surgery and global health before and after 2015.

We found no results for the GS French and Spanish queries. The year 2021 had the highest GS annual interest (8.0%), while 2020 had the highest GH interest (58.0%) (Fig. 1). The mean monthly GS interest was 1.6% (95% CI [1.4%, 1.9%]). From January 2004 to May 2021, there were 21 months (1.0%) during which the interest level was 0%, and all were before 2015. The mean GH interest level was 45.3% (95% CI [44.2%, 46.5%]). The mean difference between GS and GH increased after 2015 (45.4%, 95% CI [43.5%, 47.3%] vs. 42.9%, 95% CI [41.7%, 44.3%], P = 0.04) (Fig. 2). Additionally, the GS and GH interest levels were positively and weakly correlated (Correlation coefficient = 0.14, P = 0.04).

**Fig. 3:**
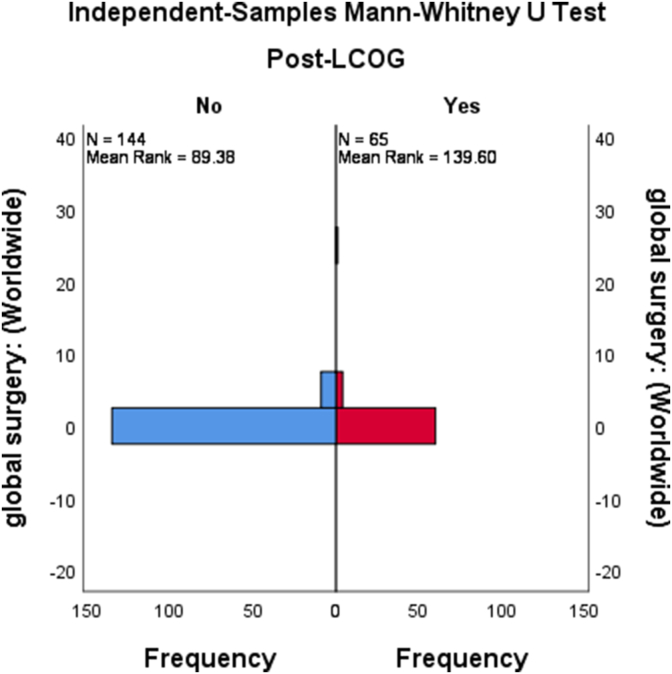
Popularity of global surgery before and after 2015.

The GS interest levels increased after 2015 (2.3%, 95% CI [1.6%, 3.0%] vs. 1.3%, 95% CI [1.2%, 1.5%], P < 0.001) (Fig. 3).

The ten countries most interested in GS were India (5.0%), the United Kingdom (5.0%), Ireland (4.0%), the United States (4.0%), Australia (3.0%), Canada (3.0%), New Zealand (3.0%), Germany (2.0%), South Africa (2.0%), and Nigeria (1.0%).

## 4 Discussion

The present study analyzed GS's global geographical and temporal expansion using Google Trends metadata to quantify interest levels. The mean monthly GS interest level was consistently lower than GH's interest levels, and GS interest levels increased significantly after 2015. Furthermore, we found that most of the ten countries most interested in GS were HICs.

A growing interest in GS is apparent in the current cohort of medical students, with students increasingly engaging in advocacy work, joining GS programs, and engaging in research [15]. This recently marked interest is a key component of social media, which has exposed students to GS inequities and initiatives where respective medical institutions have fallen short [16]. Several initiatives have been integral to this growth, such as InciSioN and Global Surgery Student Alliance (GSSA) in the United States [8,15]. Given the peaked interest, several important steps are needed to maintain said curiosity and ensure sustainable, important output. Firstly, there is an urgent need for robust, quality-assured GS curriculums to ensure basic competencies in GS are met. Courses such as that developed by Harvard Medical School and the University of Zimbabwe can be utilized to fulfill the need for a robust GS curriculum [17]. Secondly, increasing exposure to GS advocacy and research opportunities through conferences and continued dissemination of information through social media will allow students to become accustomed to necessary GS etiquette [18,19]. Current advocacy efforts include the international GS day celebrations from May 24 to 27 this year. Medical student members of InciSioN contributed significantly to GS day by disseminating posters and videos, hosting a journal club session, and organizing a Twitter chat. Medical students are an undeniable resource in global health equity, with increased downtime to dedicate to furthering the GS cause. Furthermore, medical students have unique perspectives on the future of GS [20]. Thus continuing encouragement of their participation is of utmost importance for this growing academic field.

GS research has helped advance objectives for equitable SOTA worldwide by providing data for advocacy and attracting aspiring and early career researchers. The practice of GS research has been dubbed academic GS. Academic GS is defined as the subset of GS devoted to the training of surgical educators and the discovery of new knowledge to correct the global disparity in access to surgical care [21]. Academic GS comprises clinical, educational, and research collaborations aiming at improving surgical care between academic surgeons in HICs and LMICs as well as their affiliated academic institutions [22]. GS has been emerging as an academic pursuit for a decade now. Financial investments are being made to establish sustainability in the mission of academic GS development. However, the inclusion of GS remains in its infancy. Although academic GS has been facing hardships, it is evolving to become a groundswell of interest among medical students, residents, and faculty [23,24]. Four years have passed since the Global Academic Surgery Committee of the Society for University Surgeons published a guide to research in GS, and several studies have been published on the subject since then [25]. Inequity in access to surgical care is the core determinant in the rise of global academic surgery as a field. However, there are few opportunities for surgery residents and trainees to be involved in GS [21,26]. Indeed, a survey by Abraham et al. noted that few general surgery programs disposed of an international training program (17%), and only a few of them offered mixed research and clinical opportunities [26]. It is evident that the emerging field is less known and addressed in LMICs contrary to HICs, where the field is at the center of all attention. A review by Park et al. noted that in the last decade, only 15% of studies addressing GS issues were conducted in LMICS. Most of them were performed in sub-Saharan Africa (Ghana, Uganda, Ethiopia, and Tanzania) [22]. To develop academic GS, several approaches have been tested. Among them, we cite global research fellowship, GS elective programs, and advocacy initiatives [21,22,26,27]. Despite the existence of several electives, few opportunities exist for surgery residents in LMICs. Most of the opportunities being offered in LMICs are reserved for HICs residents, a disparity of opportunities that must be addressed urgently [26].

Even though LMICs need better health care services, the level of interest shown by LMICs towards GS according to the results generated in this study reflects the state of GS in LMICs. Physicians from HICs visit LMICs to work with local physicians to strengthen GS capacity in such regions; however, Alyssa Scheiner et al., in their findings, noted that this could be another form of colonialism LMICs by HICs [28]. If true, this form of colonialism might be the reason for hesitancy by local surgeons to collaborate effectively with their HICs counterparts. Although GS began to gain recognition globally in 2015 after the LCoGS report and WHO resolution, the level of growth and implementation of GS in LMICs is still disproportional to the unmet surgical burden besieging these regions yearly, likely due to the low level of participation in GS seen among physicians in LMICs. Global health conferences could be a platform for LMICs physicians to gain more diverse perspectives and connect with physicians from HICs for a more collaborative effort towards fostering GS capacity in their respective regions. Unfortunately, physicians from LMICs are minimally represented in the GH conferences due to systemic barriers covering high travel costs, visa restrictions, and lower acceptance rates for research presentations [29]. In a bid to advance access to good surgical care in Rwanda, the GS Unit Hub in Rwanda has been working with local surgeons, medical students, public bodies, and communities to achieve surgical equity [30]. More GS researchers in LMICs through collaboration with researchers in HICs would help reposition GS in LMICS as seen in Rwanda [7].

Since our study uses Google Trends, it is possible LMICs are underrepresented due to the lower internet coverage in these regions. However, considering that the internet has been the primary mode of communication and advocacy for GS, this study accurately reflects popularity in countries with greater internet coverage. Further, Google Trends uses Google Search metadata, excluding queries from other popular search engines like Bing (Microsoft, WA, USA). Google Search dominates the global search engine market boasting almost 90% of the market share.30 As such, the omitted queries on the other search engines are within an acceptable margin of error. Finally, traditional media coverage has been shown to confound search patterns [12]. Hence, regions and periods with greater media coverage can bias search queries without necessarily reflecting a direct relationship between interest levels and a particular time or region. Notwithstanding, we believe our study gives valuable insight into the popularity of GS worldwide since 2004.

## 5 Conclusion

The present study revealed higher GS interest levels than Global Health, especially in HICs with further increased interest levels post-2015 Lancet Commission seminar report on GS and the World Health Organization resolution WHA 68.15. Despite those seminal publications, the GS interest levels in LMICs are still not proportional to the unmet surgical burden those countries are currently facing. Therefore, subsequent advocacy efforts should focus on LMICs to expand GS worldwide, ensuring all countries can reach the GS 2030 targets and reduce the global burden of surgical diseases.

## Provenance and peer review

Not commissioned, externally peer-reviewed.

## Ethical approval

No ethical approval was needed for this research study.

## Sources of funding

No funding was received for this study.

## Author contribution

Lorraine Arabang Sebopelo: Analysis and interpretation of data; Drafting of manuscript; Critical revision; approved the final version.

Alexandre Jose Bourcier: Analysis and interpretation of data; Drafting of manuscript; Critical revision; approved the final version.

Olaoluwa Ezekiel Dada: Drafting of manuscript; approved the final version.

Gideon Adegboyega: Drafting of manuscript; approved the final version.

Daniel Safari Nteranya: Drafting of manuscript; approved the final version.

Ulrick Sidney Kanmounye: Study conception and design; Acquisition of data; Analysis and interpretation of data; Drafting of manuscript; Critical revision; approved the final version.

## Registration of research studies

1. Name of the registry: Clinical Trials Protocol Registration and Results System.

2. Unique Identifying number or registration ID: NCT05012085.

3. Hyperlink to your specific registration (must be publicly accessible and will be checked): https://clinicaltrials.gov/ct2/show/NCT05012085.

## Guarantor

All three the authors (LAS, AJB, OED, GA, DSN, USK) accept full responsibility for this study and manuscript.

## Declaration of competing interest

The authors do not have any conflicts of interest to disclose.
